# Response to Commentary: *Arnica montana* Effects on Gene Expression in a Human Macrophage Cell Line. Evaluation by Quantitative Real-Time PCR

**DOI:** 10.3389/fimmu.2016.00320

**Published:** 2016-09-09

**Authors:** Debora Olioso, Marta Marzotto, Clara Bonafini, Maurizio Brizzi, Paolo Bellavite

**Affiliations:** ^1^Department of Medicine, University of Verona, Verona, Italy; ^2^Department of Statistical Sciences, University of Bologna, Bologna, Italy

**Keywords:** *Arnica*, wound healing, gene expression regulation, macrophages, inflammation

We recently investigated the effects of *Arnica montana* (*Arnica*) on the THP-1 myelomonocytic cell line, differentiated by phorbol-myristate acetate and IL-4 in the wound healing phenotype (M2) (1). Our study was the object of a commentary by Chirumbolo and Bjørklund (2), based on some recalculations and on their extrapolations from the values of SEM. Even though we had correctly reported the dosage of sesquiterpenes – reference substances for *Arnica* pharmacopeia – the authors inferred that this information was insufficient and they recalculated the doses in terms of helenalin: “in the starting 30% alcoholic preparation of *Arnica* (1c), a percentage of 0.036% sesquiterpene lactones, should correspond to 0.72 μg, i.e., 72 ng in the dilution 2c.” This calculation is flawed because our dose was referred to the mother tincture and not to the 1c dilution. Moreover, helenalin is not the only active ingredient in *Arnica*, which contains many other substances (3, 4). Finally, 0.72 μg in 1c are not 72 ng in 2c but 7.2 ng.

Since a cellular target of helenalin is NF-κB, the authors argue that we did not show any effect “despite the existence of further reports published elsewhere showing an effect of sesquiterpene lactones on NF-κB.” Actually, it is true that we found little effect on NF-κB gene expression, but the cited effects of helenalin (2) reported elsewhere were not due to gene downregulation but to the inhibition of protein (that we did not test). The authors then proceed to evince skepticism that our results were obtained “in dilutions with active principles very far from a pharmacological bioactivity and without an apparent dose–response behaviour.” However, data that do not fall within linear dose–response relationships are not at all uncommon in pharmacology and immunology, and they result from various possible causes (5).

In the central part of the commentary (2), Chirumbolo and Bjørklund state that “showing only SEM or other parameters excluding raw data cannot allow the reader to realize about any statistical reliability and reproducibility.” Then, they claim to have “evaluated statistic distribution of SEM variability to ascertain if any reported outliers due to bias error affected the behaviour of the investigated samples.” The inherent contradiction in these two statements is easy to detect.

The authors do not have access to the original dataset, which – as almost always happens in scientific articles – was not reported by Olioso et al. (1) In fact, they only saw the exploratory statistics tables from which they tried to extrapolate the distribution of the values of SEM, by pretending to find outliers there. Well, this procedure is actually an outlier in statistical practice! The different values of SEM are clearly not a sample of observations, and each of them refers to a different gene from cells in different conditions, so studying the distribution of SEM values is totally meaningless. When working with small samples (here we have sizes from *n* = 6 to 10), an observation can be considered as an outlier only when it is greatly divergent from the others. According to the Dixon *Q*-test, the distance between the observation itself and the nearest one has to be equal or larger than one half of the total range. This would become apparent immediately just by reading the data carefully, and it should go without saying that such a check has been carried out on the dataset: in fact, for each treatment we checked the statistical homogeneity, the global comparison by means of ANOVA, the level of skewness, and the result of the *T*-test (see Methods) (1).

The commentary (2) contends that we “did not report reproducible data for the highest concentrated dilution used in their study, i.e., *Arnica* 2c, which appeared to exhibit a significant action quite only on THP-1 cell line following activation with IL-4.” This concept is misleading because our cells were profoundly changed by IL-4 treatment as shown by marker genes and the better effects of *Arnica* in these cells was a key finding of our investigation. So, looking for “reproducibility” in different cell types, as they have tried with a “linear regression” between M0 with M2 macrophages in their figure 1 (2), is misrepresentative of our approach.

Some sentences (2) are beyond any understanding: “Probably, cells undergoing less stress (24 h incubation at 37°C 5% CO_2_) and addressed to drive any molecular machinery to a highly controlled response to a stimulus (i.e., IL-4) amplified signals vs. noises, i.e., reduced the difference between noises and statistical variability.” Equally, vague is the phrase “intra-assay and inter-assay variability, expressed as SEM, contained possible bias due to the dispersion effect of the reported variability and outlying data.” It is not clear how the authors can presume that the data variability could contain “bias.” The existence of genes with different stability is well known to experts on molecular biology and specifically on gene expression. We reported that even housekeeping genes have different variability (1). In any case, the fact that some macrophage genes in some conditions were characterized by a high experimental variability is not surprising, because the size of the effect was generally small, probably due to the low dose of the drugs used. Moreover, it is quite strange that the authors (2) have some doubts about the correct use of a non-parametric test (Wilcoxon test, specifically); it should be definitively undisputed that, when working with skewed data, it is not possible to apply standard parametric tests.

We agree that by replicating the data, one could better appreciate the effects of *Arnica* on inflammatory gene upregulation. The whole field of high-dilution pharmacology needs further investigative efforts and experimental contributions; however, this certainly does not constitute a criticism of our work. Our findings provided a first albeit preliminary molecular explanation for the effects of *Arnica* on macrophages.

## Author Contributions

PB and MB wrote the text after extensive discussion with all the authors.

## Conflict of Interest Statement

The authors declare that the research was conducted in the absence of any commercial or financial relationships that could be construed as a potential conflict of interest.
